# Energy-coupling mechanism of the multidrug resistance transporter AcrB: Evidence for membrane potential-driving hypothesis through mutagenic analysis

**DOI:** 10.1007/s13238-017-0417-3

**Published:** 2017-05-11

**Authors:** Min Liu, Xuejun C. Zhang

**Affiliations:** 1 National Laboratory of Biomacromolecules, CAS Center for Excellence in Biomacromolecules, Institute of Biophysics, Chinese Academy of Sciences, Beijing, 100101 China; 2 University of Chinese Academy of Sciences, Beijing, 100049 China

**Dear Editor,**

RND (resistance nodulation-cell division) exporters are widely expressed across the lineage of Gram-negative bacteria (Fig. S1). Their functional role is to utilize the proton-motive force of the cell to drive the expulsion of cytotoxic substances from the outer leaflet of the inner membrane as well as the periplasm to extracellular space (Yamaguchi et al., 2015). Extensive structural studies on RND transporters have provided significant insights into mechanisms of substrate transport. For example, the *E*. *coli* RND transporter AcrB (EcAcrB) forms a homo-trimer (Murakami et al., 2002), which further forms a complex with an outer-membrane conduit formed by TolC as well as adaptor proteins (AcrA) (Jeong et al., 2016). The AcrB trimer consists of three layers, namely transmembrane (TM) layer, porter layer, and adaptor-docking layer (Fig. 1). In complex structures with substrates, each of the three protomers of the AcrB trimer assumes a distinct conformation (Murakami et al., 2006; Seeger et al., 2006). Thus, a functional cycle of AcrB transport consists of three phases, termed as access, binding, and extrusion phases. One important mechanistic question to address for RND transporters then is how substrate binding in the porter layer induces proton transport in the TM layer, which in turn is required to drive the transport of substrate across the outer membrane.

**Figure 1:**
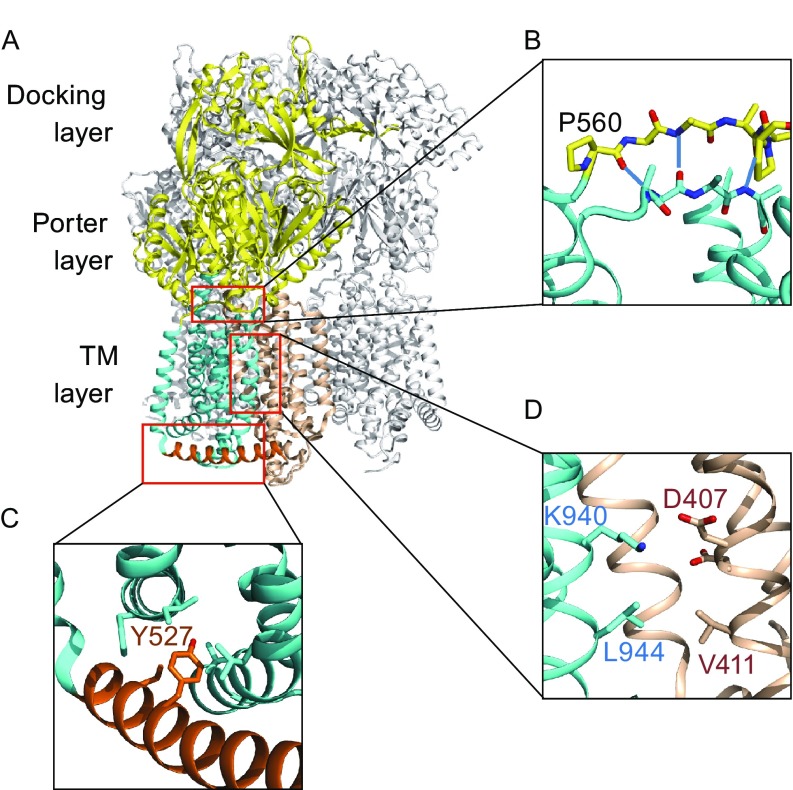
**AcrB structure and mutation sites**. (A) Trimer crystal structure of *E*. *coli* AcrB (PDB ID: 4DX5). One protomer is shown in color, and the other two in grey. The N_TM_ and C_TM_ subdomains are shown in wheat color and cyan, respectively. The porter domain and docking domain are in yellow. The cytosolic-side amphipathic helix, α6-7, is colored in orange. (B) The signaling motif-C in C_TM_. The three main-chain H-bonds are depicted as dash-lines. Except proline residues, side-chains are omitted for clarity. (C) The α6-7 region. (D) Region of the titratable key residue(s)

The structure of an AcrB protomer shows a pseudo two-fold symmetry (Murakami et al., 2002). The TM domain contains 12 TM helices, forming two subdomains termed as N_TM_ (TMs 1–6) and C_TM_ (TMs 7–12). A group of conserved residues, including D407^TM4^ (i.e., Asp407 in TM4), D408^TM4^, and K940^TM10^, form a water molecule-mediated hydrogen-bond (H-bond) network in the subdomain interface (Fig. 1D) (Eicher et al., 2014). Two large inserts exist between TMs 1 and 2, as well as between TMs 7 and 8. The insert in N_TM_ forms subdomains PN1 and PN2 of the porter domain, while the insert in C_TM_ forms subdomains PC1 and PC2. The substrate-transport path in the porter domain is located between PN1, PN2, PC1, and PC2 (Yamaguchi et al., 2015). In its extrusion phase, the TM domain of an EcAcrB protomer assumes a different conformation from the other two protomers (Sennhauser et al., 2007). More specifically, in the extrusion phase, K940^TM10^ is located more distantly from D407^TM4^ and D408^TM4^, thus one of these acidic residues is likely to become protonated. Once one of the two acidic residues becomes protonated, the p*K*_*a*_ of the other will decrease. In contrast, in the other two phases, the positive charge of the K940 side-chain moves in between side-chains of D407 and D408, and thus neither of the two acidic residues is likely to be protonated. Therefore, configurational change of this H-bond network affects the p*K*_*a*_ of the proton-titratable, key residue D407^TM4^ (which is more conserved than D408), thus affecting proton transport (Yamaguchi et al., 2015).

In the present work, we used single-point mutagenesis in combination with functional complementation assays on the prototypic RND transporter, EcAcrB to test a membrane potential-based hypothesis on the energy-coupling mechanism of RND transporters. In the complementation assay, we used the *acrb-*knockout, *E*. *coli* strain MG1655, referred to as Δ*acrb* hereafter, as the background strain, which is sensitive to several antibiotics, including to ciprofloxacin. As a positive control, wild type (WT) EcAcrB expressed in the Δ*acrb* cell by using a pET21a-based plasmid was able to rescue the cell growth in the presence of 5-ng/mL concentration of ciprofloxacin (Fig. 2A). In contrast, the empty vector of pET21a was unable to rescue the cells. In addition, D407N (Asp407 to Asn mutation), previously shown to be transport-incompetent in homolog transporter MexB (Guan and Nakae, 2001), was also used as a negative control. All point mutations as well as WT EcAcrB used in this study were expressed as C-terminal His6-tagged proteins. Their expression levels were confirmed using immunoblotting against His-tag (Fig. S2A). Selected AcrB variants were further subjected thermofluor analyses (Cummings et al., 2006) to confirm that their thermostabilities were comparable to that of WT (Fig. S2B).

**Figure 2:**
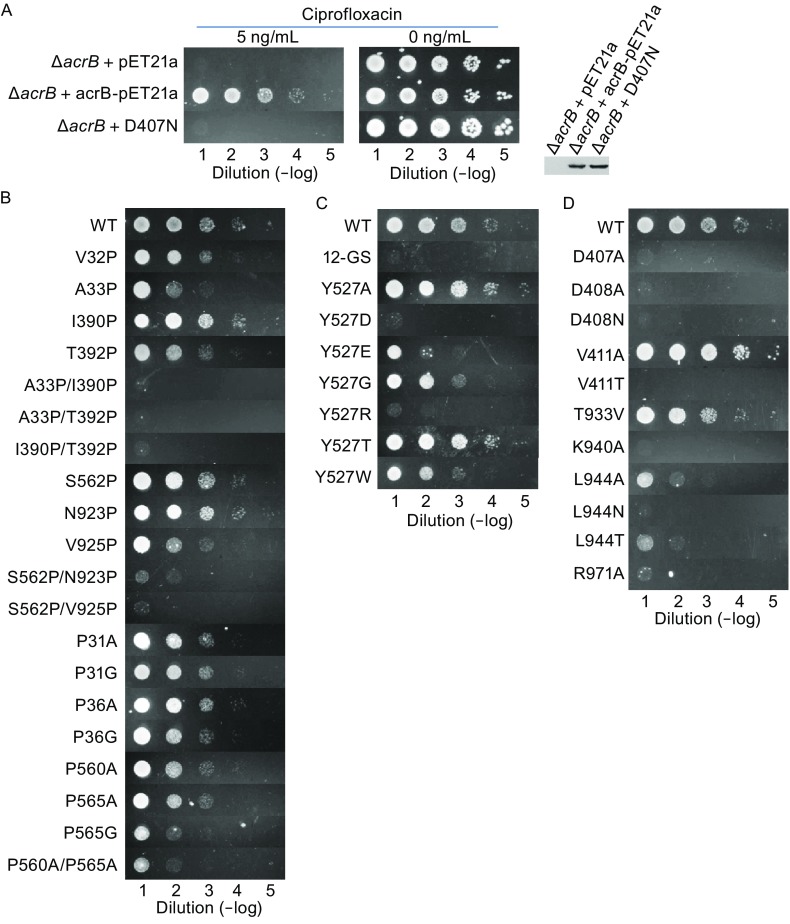
**Anti-ciprofloxacin complementation assay**. (A) A serial dilution of cell culture spotted onto solid medium containing 100 µg/mL ampicillin and 5 ng/mL ciprofloxacin. Compared with the empty-vector (pET21a) and predicted protonation-site mutant D407N containing strain, the wild type AcrB constructed in pET21a showed apparent resistance against ciprofloxacin. Leaky expression of AcrB was confirmed using anti-His immunoblotting. (B) Mutational effects in the two signaling motif regions. (C) Mutational effects in the amphipathic helix α6-7. 12-GS is the variant that the entire α6-7 was replaced with 12 Gly-Ser repeats. (D) Mutational effects in the region of titratable key residues

Firstly, we studied the communication mechanism between the porter layer and TM layer. The subdomain N_TM_ contains a β-sheet (termed to as signaling motif-N) consisting of one strand (strand-N1: residues P31–P36) connecting the helix TM1 with the PN1 subdomain of the porter domain and another strand (strand-N2: residues I390–T392) connecting TM3 with TM4. Similarly, the subdomain C_TM_ contains a β-sheet (signaling motif-C, Fig. 1B) consisting of one strand (strand-C1: residues P560–P565) connecting TM7 with the PC1 subdomain and another strand (strand-C2: residues N923–V925) connecting TM9 with TM10. To test the interdomain communicating function of the two short parallel β-sheets, we disrupted the β-sheet formation by introducing point mutations at different positions in the four β-strands.

Within the signaling motif-N, three main-chain H-bonds are formed between P31-O and I390-N; between A33-N and I390-O; and between Q34-O and T392-N. Among the point mutations in signaling motif-N, double point mutations A33P/I390P and I390P/T392P showed drastically reduced ability to complement *∆acrb* in the presence of ciprofloxacin (Fig. 2B). The single point mutations A33P and T392P also resulted in slightly reduced transport activity, whereas the I390P variant maintained WT activity. Similarly, in the signaling motif-C, three main-chain H-bonds are formed between P560-O and N923-N; between S562-N and N923-O; and between F563-O and V925-N. The double point mutant forms S562P/N923P and S562P/V925P lost all activity. The V925P variant exhibited partially reduced drug resistance activity, whereas the mutations S562P and N923P failed to show detectable reduction in activity. In addition, the mutation V32P within the signaling motif-N (at a position not directly involved in H-bonding) also resulted in slightly reduced activity, presumably due to effects on the backbone conformation and/or flexibility of the β-strand. Together, these data indicated that disrupting two of the three H-bonds in each signaling motif is sufficient to abolish the transport activity of AcrB. Another characteristic feature of the signaling motifs is the fact that the first β-strand in each β-sheet is flanked by two proline residues (Fig. S1). In the signaling motif-C, mutations P565G and P560A/P565A showed drastically reduced drug-resistance activity, while activities of other mutations at the flanking proline residues reduced slightly. Taken together, these results suggest that the interdomain β-sheets play important roles in the communication between the porter and TM domains.

Secondly, to verify the functional importance of the long amphipathic helix (α6-7, residues G511–S537, between TM6 and TM7), we first replaced this helix with 12 repeats of Gly-Ser pair (12-GS). As shown in Fig. 2C, this variant completely lost its drug-resistance activity. In addition, Y527 (a conserved residue in α6-7, Fig. S1) was mutated to a variety of amino acid residues. It appeared that variants containing a charged residue at this position, Y527D (E) and Y527R, lost the transport activity. The activities of Y527G and Y527W were found to be slightly reduced, whereas the mutant variants Y527A and Y527T maintained their activity. Together, these results suggest that α6-7 not only serves as a structural linker between N_TM_ and C_TM_, but also plays important functional roles, more likely serve as a pivotal point for the electrostatic-force generated mechanical torque (Zhang et al., 2014; Zhang et al., 2015). In addition, the conserved residue Y527 may be involved in anchoring the α6-7 to N-terminal of the C_TM_ subdomain (Eda et al., 2003), which is important to the transport activity.

Thirdly, we studied the proton wires associated with transport activity. We found that in the protonation status, the downstream proton wire is likely to be blocked by a cluster of conserved hydrophobic residues, including V411^TM4^ and L944^TM10^, to prevent proton leakage (see the protomer C in the 1.9-Å resolution structure of EcAcrB, PDB file 4DX5). This hydrophobic blockage is presumably removed in the deprotonated status due to the relative movement between TMs 4 and 10. In agreement with their putative roles in the hydrophobic blockage, point mutations of V411T (low expression), L944N, and L944T resulted in loss of transport activity (Fig. 2D) (V411N failed to be expressed). Moreover, while L944A lost the activity, V411A maintained its activity. Their difference in activity is presumably related to the difference in length of their truncated hydrophobic side-chains. In addition, in agreement with previous reports (Guan and Nakae, 2001; Takatsuka and Nikaido, 2006; Seeger et al., 2009), point mutations D407A, D407N, D408A, D408N, K940A, and R971A abolished the activity.

In contrast to many other secondary active transporters, the substrate-binding porter domain and the proton-transport TM domain of RND transporters are structurally separated (Murakami et al., 2002). It is therefore paramount for the function of RND transporters that an effective signaling pathway is established between the two domains. The two functionally distinct domains are connected through four β-strands, which are involved in β-sheet formation at the interface between the porter- and TM-layers. These β-strands are likely to serve as signaling structural elements. More importantly, the PC1 subdomain has been shown to be involved in a large conformational change upon substrate binding (Sennhauser et al., 2007). Our mutagenic analysis showed that disrupting either of these two β-sheets results in either reduced or abolished transport activity (Fig. 2B).

Upon protonation, the proton-titratable, key residue D407^TM4^ positioned in the middle of the TM domain is subjected to an additional electrostatic force (Fig. S3). Together with hydrophobic mismatch forces (Mouritsen and Bloom, 1984), this electrostatic force generates a mechanical torque and is likely to result in tilting of the TM domain. Through the connecting peptides, this tilting movement of the TM domain relative to the membrane is likely to drive the conformational change inside the porter domain and results in substrate transport. Previously, it has been shown that many mutations in the H-bond network around the protonation site, e.g., at positions D407, D408, K940, and T978, result in reduced transport activity. In the present work, we further showed that properly switching off the proton wire connecting to the cytosolic side is essential for EcAcrB transport activity. Disrupting the switching process (e.g., L944N) abolishes transport activity (Fig. 2D). Furthermore, the electrostatic force exerted on the titratable key residue D407^TM4^ does not pass through the amphipathic helix α6-7 (which is attached to the cytosolic surface of the membrane). Thus, this helix is expected to serve as a pivotal point to generate the rotation torque required for tilting of the TM domain when protonation occurs. Our mutagenesis data showed that disrupting the amphipathic property of α6-7 or its interaction with the TM domain reduces or abolishes the transport activity of EcAcrB (Fig. 2C).

Because the two interdomain β-sheets of AcrB, together with its helix α6-7 are distally located to the known substrate binding sites as well as to the transport path inside the porter domain, the effects of their mutational changes on transport activity are unlikely to be directly related to substrate binding. On the basis of our results, we propose the following model for the AcrB transport: First, substrate-binding signal from the porter domain induces a relative movement between TMs 4 and 10. Second, this movement results in the protonation of the key titratable residue D407^TM4^. Third, the protonated TM domain is subjected to an inward electrostatic force from the negative-inside membrane potential. Lastly, this force converts the electrostatic energy of the proton to mechanical energy, which in turn drives the transport process of the substrates.

## **FOOTNOTES**

The authors thank Dr. Yong Tao of the Institute of Microbiology, CAS, for providing the ∆*acrB* cell strain. We thank Dr. Torsten Juelich for linguistic assistance during the preparation of this manuscript. This work was supported by the National Basic Research Program (973 Program) (No. 2015CB910104 to XCZ) and National Natural Science Foundation of China (Grant No. 31470745 to XCZ).

Xuejun C. Zhang and Min Liu declare that they have no conflict of interest. This article does not contain any studies with human or animal subjects performed by the any of the authors.

## Electronic supplementary material

Below is the link to the electronic supplementary material.

Supplementary material 1 (DOCX 940 kb)
