# Supplementary material for: Energy-coupling mechanism of the multidrug resistance transporter AcrB: Evidence for membrane potential-driving hypothesis through mutagenic analysis
Source: Protein Cell. 2017 May 11;8(8):623–7. doi: 10.1007/s13238-017-0417-3 (PMC5546932; doi:10.1007/s13238-017-0417-3)
Supplement: Supplementary file 1 — Supplementary material 1 (DOCX 940 kb) [file 13238_2017_417_MOESM1_ESM.docx]

# Supplementary materials

## Material and methods

The full length *E. coli* *acrB* gene was cloned into pET21a, which expresses AcrB with a C-terminal His_6_-tag. Mutation variants were constructed using the overlap-PCR method, and mutation success was confirmed by DNA sequencing. The constructs were transferred into an *acrB*-knockout strain of MG1655, referred to as Δ*acrB*. Liquid LB containing ampicillin (100 µg/ml) was then inoculated with a single colony and cultured at 37°C. At OD_600_ reaching 0.5–0.8, cells were centrifuged and resuspended in LB to adjust OD_600_ to 1.0. Suspension of each construct was further diluted 10 to 100,000 fold. A series of dilutions (1 µl each) were spotted onto a solid LB medium supplemented with 100 µg/ml ampicillin and 5 ng/ml ciprofloxacin. After 12 h incubation at 37°C, colony formation was recorded to verify the ciprofloxacin resistance of each mutation variant. Since the cell strain MG1655 is not compatible with IPTG induction, drug resistance of AcrB variants constructed in pET21a against ciprofloxacin was the result of leaky expression in the absence of IPTG. The membrane fraction of cell lysates was collected, and the leaky expression levels of AcrB variants were estimated by using anti-His immunoblotting.

## Supplementary figures


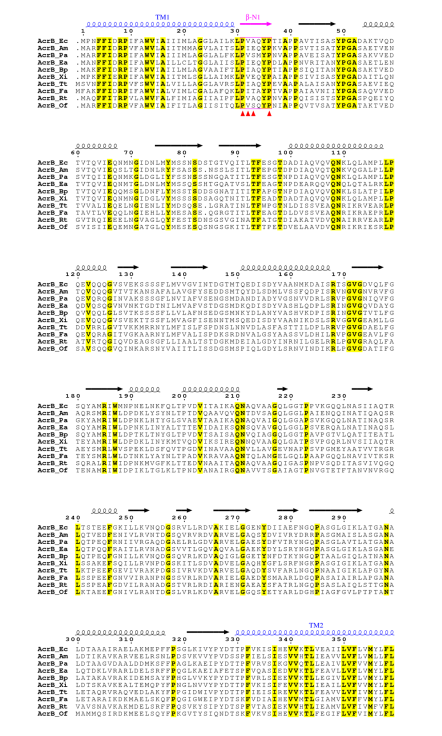

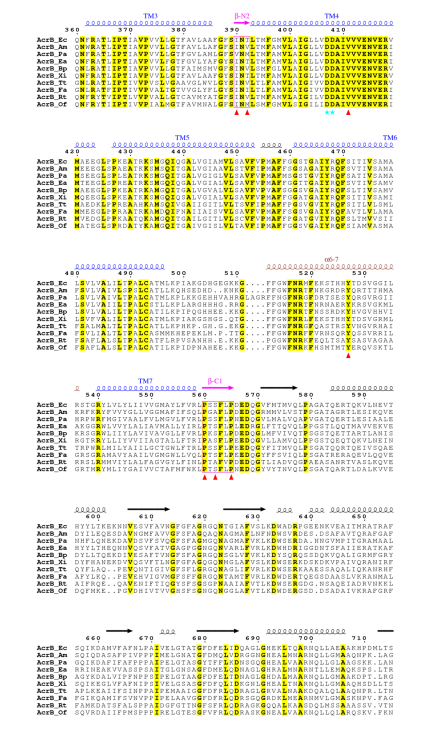

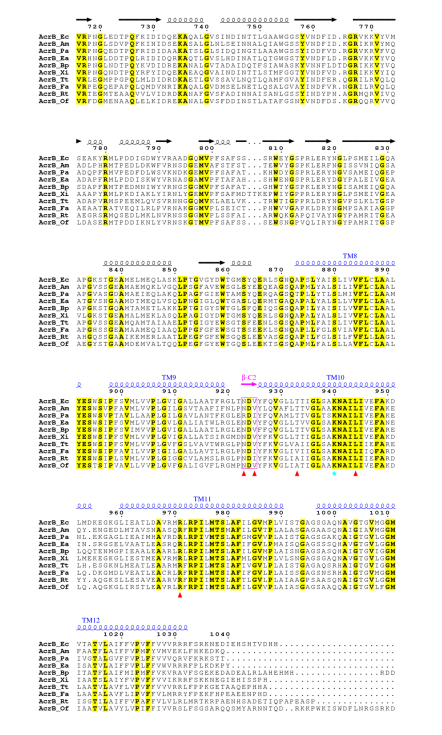


## Figure S1. Sequence comparison of AcrB homologs

Amino acid sequence alignment of AcrB from E. coli (AcrB_Ec), Alteromonas mediterranea (AcrB_Am), Pseudomonas aeruginosa (AcrB_Pa), Pseudomonas aeruginosa (AcrB_Pa), Erwinia amylovora (AcrB_Ea), Burkholderia pseudomallei (AcrB_Bp), Xenorhabdus innexi (AcrB_Xi), Tepidiphilus thermophilus (AcrB_Tt), Ferriphaselus amnicola (AcrB_Fa), Rhizobium tropici (AcrB_Rt), and Oxalobacter formigenes (AcrB_Of). Secondary structural elements were sourced from AcrB_Ec (PDB file: 4DX5), and are marked on the top of the aligned sequences. The transmembrane domains (TM) are in blue, and strand-N1 (β-N1), strand-N2 (β-N2), strand-C1 (β-C1), and strand-C2 (β-C2) are indicated in pink and grouped in pink boxes. Residues that are known to be involved in protonation are indicated with cyan stars, and other residues that are mutated for complementation assay are indicated with red triangle. The sequence alignment was performed with Clustal Omega and formatted with ESPript 3.


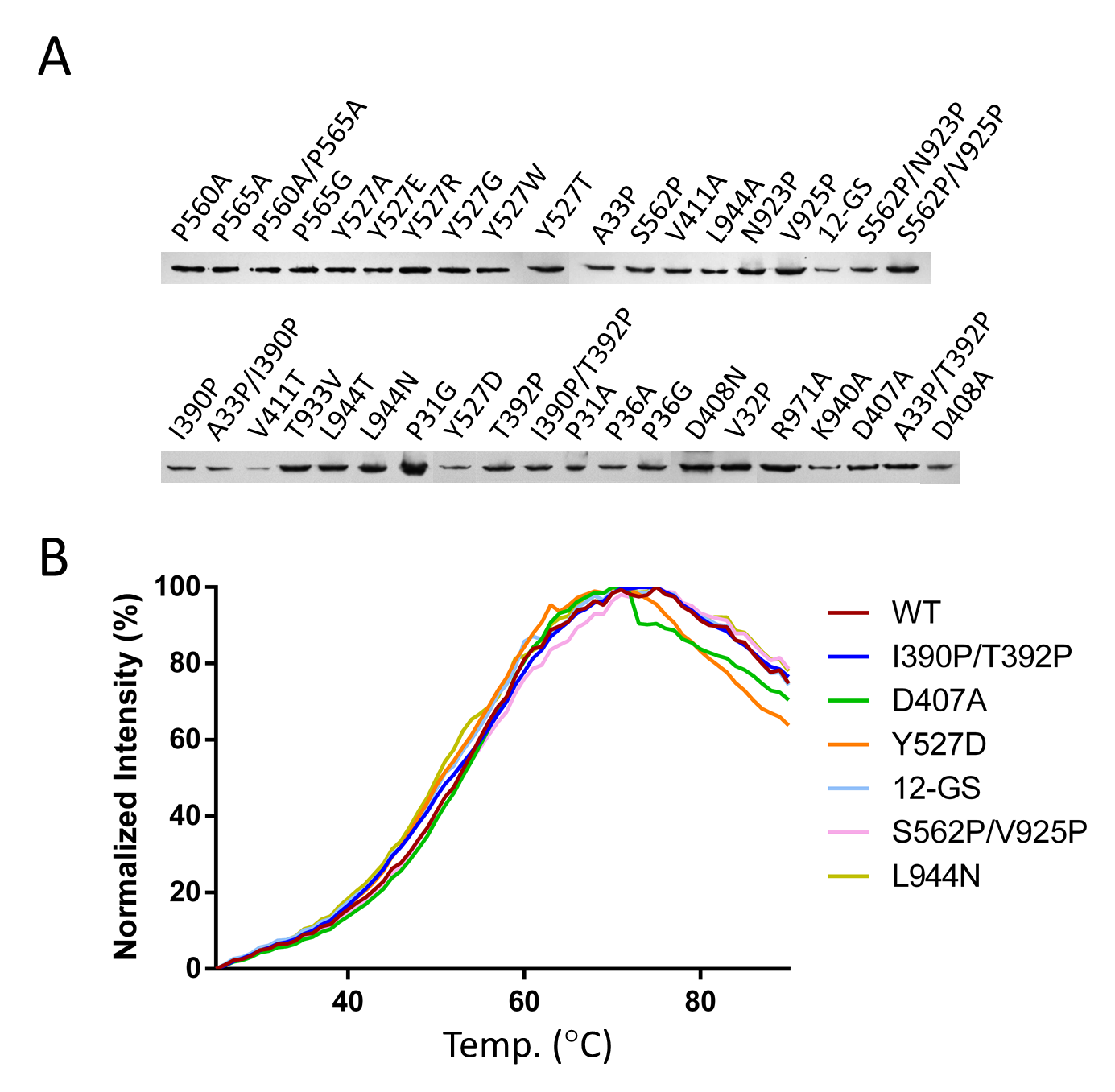


## Figure S2. Analyses on AcrB variants

A. Confirmation of the expression of AcrB variants. Cell cultures of each variant were harvested, lysed by sonication, and centrifuged at 12,000 g for 10 min. The supernatants were then transferred to a new microcentrifuge tube, and cell membrane fractions were pelleted at 17,000 g for 40 min. The pellet fraction was subjected to SDS PAGE electrophoresis. Proteins were transferred onto nitrocellulose blotting membrane (GE Healthcare, Germany) and immunoblotted with anti-His antibodies (CWBIO, China). B. Thermofluor analysis of selected AcrB variants. The assay was performed using a qPCR instrument (Rotor-Gene 6600, Corbett Research, Australia). As temperature rose, thiol-specific fluorochrome N-[4-7- (diethylamino-4-methyl-3-coumarinyl)phenyl]maleimide (CPM from Invitrogen, US) was used as fluorescence probe to monitor the conformational change. CPM fluorescence signal was measured with a 387 nm excitation and a 463 nm emission. The purified protein of WT EcAcrB or its variants were added into reaction buffer (100 mM TRIS (pH 8.0), 100 mM NaCl, 25 μM CPM, and 0.5 mM n-Dodecyl-β-D-Maltoside). Reaction volume was 25 μl, and the final protein concentration was 0.4 μg/μl. The qPCR instrument was programed to increase temperature by 1°C in 1 min and then to stay at each temperature for 1.5 min between 25°C and 90°C. Data analyses were performed using the program Graphpad Prism.


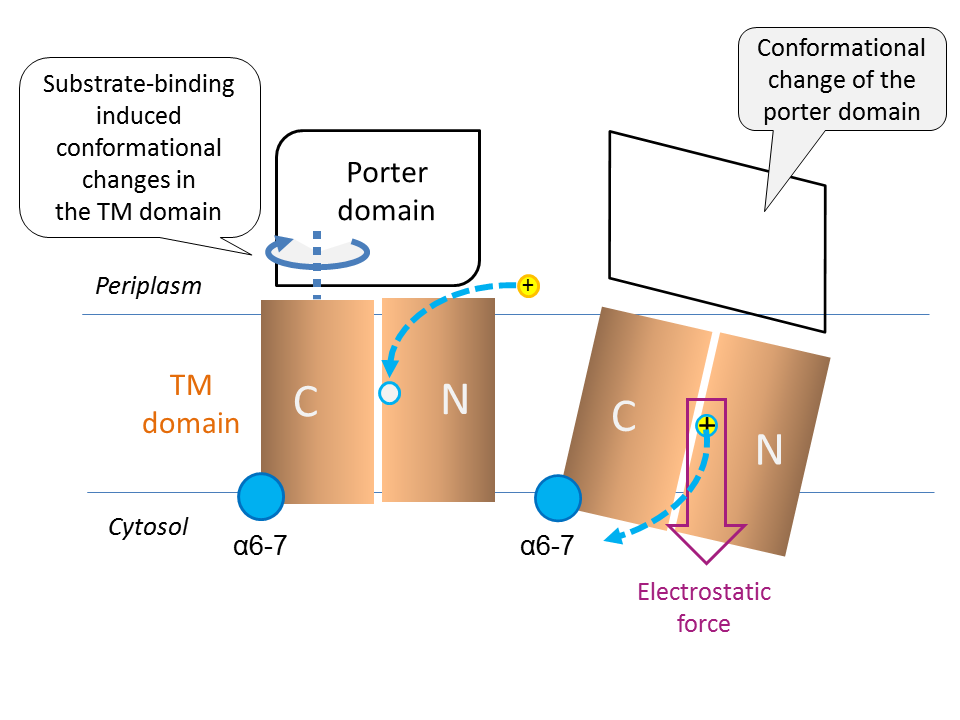


## Figure S3. Schematic diagram of energy-coupling between the TM and porter domains of RND transporter
